# From basic perception deficits to facial affect recognition impairments in schizophrenia

**DOI:** 10.1038/s41598-019-45231-x

**Published:** 2019-06-20

**Authors:** Csilla Marosi, Zsuzsanna Fodor, Gábor Csukly

**Affiliations:** Department of Psychiatry and Psychotherapy, Semmelweis University, Budapest, Hungary

**Keywords:** Emotion, Visual system

## Abstract

While impaired facial emotion recognition and magnocellular deficits in visual perception are core features of schizophrenia, their relationship is still unclear. Our aim was to analyze the oscillatory background of these processes and to investigate the connection between the magnocellular pathway deficit and the abnormal facial affect processing. Thirty-nine subjects with schizophrenia and forty socially matched healthy controls subjects were enrolled. A 128 channel EEG was recorded in three experimental tasks: first, participants viewed magnocellular biased low-spatial frequency (LSF) and parvocellular biased high-spatial frequency (HSF) Gabor-patches, then faces and houses were presented and in the third task a facial affect recognition task was presented with happy, sad and neutral faces. Event-related theta (4–7 Hz) synchronization (ERS) (i.e. an increase in theta power) by magnocellular biased stimuli was decreased in patients relative to controls, while no similar differences were found between groups in the parvocellular biased condition. ERS was significantly lower in patients compared to healthy controls both in the face and in the emotion recognition task. Theta ERS to magnocellular biased stimuli, but not to parvocellular biased stimuli, were correlated with emotion recognition performance. These findings indicate a bottom up disruption of face perception and emotion recognition in schizophrenia.

## Introduction

Schizophrenia is a severe mental disorder characterized by positive, negative and cognitive symptoms. More than 21 million people are affected by this disorder all over the. Impaired facial emotion recognition is one of the core features in schizophrenia and related the patients’ social functioning and quality of life^1^. Also a growing literature supports an early sensory processing deficit in patients with schizophrenia^2–4^. In this study the relationship of these core features of schizophrenia were investigated.

The human subcortical visual system can be divided into two main parts, the parvocellular and the magnocellular pathway^5^. These pathways are the connection between the retina and the primary visual cortex (V1, striate cortex) via the lateral geniculate nucleus. The information transmission across the magnocellular pathway is rapid and low-contrasted, this system orients the attention to the space (‘where’ system) and projects to the dorsal visual stream. The information transmission across the parvocellular pathway is slower, but more detailed, this system is critical for object identification (‘what’ system) and projects to the ventral visual stream^6,7^. Therefore the magnocellular pathway is more sensitive to low-spatial frequency (LSF) and the parvocellular system is more sensitive to high-spatial frequency (HSF) stimuli^8^.

Several behavioral studies have reported impaired backward masking^9,10^, motion processing^2,11,12^, contrast sensitivity^13^, object recognition^14^ and reading^15^ in patients with schizophrenia. Steady-state visual-evoked potential (ssVEP)^13^ and transient visual-evoked potential (VEP)^14,16–19^ studies have demonstrated magnocellular deficit in patients with schizophrenia, reflecting in a decreased P100. Furthermore, recent fMRI investigations have found decreased activation to low-spatial frequency, but not to high-spatial frequency, which also indicate an exclusive magnocellular deficit in schizophrenia^4,20,21^.

Previous studies reported face and emotion recognition impairments in schizophrenia^22^. Since EEG has a good temporal resolution, event related potential (ERP) paradigms have long been used to study the different stages of facial emotion processing^23^. Early stage of perception can be linked to P100, the structural decoding of the face indicated by the N170 and the higher level processing of facial emotions indexed by N250 component. Several previous experiments have described deficits in these three ERP components in schizophrenia^24–28^, although some other studies have found no differences between patients and healthy controls^29–32^.

The bottom-up model of schizophrenia states that the early sensory impairments lead to higher level process deficits such as facial emotion processing, which further contribute to the psychosocial functioning impairments^33^. Early visual dysfunction and impaired emotion recognition have been observed in patients with schizophrenia, and Butler *et al*. found that emotion recognition performance was correlated with impaired magnocellular function^34^. Recent behavioral studies examined the interaction between early visual processing deficit and impaired emotion recognition via spatial frequency biased pictures of faces^35–37^. Electrophysiological studies^38,39^ found an impaired P100 component for low spatial-frequency biased fearful faces in schizophrenia. In a recent study Martinez *et al*. analyzed the correlation between visual sensory function and face emotion recognition. They showed that reduced motion sensitivity correlated with impaired face-emotion recognition in patients with schizophrenia and attenuated psychosis^40^. Taken together, previous investigations indicate that altered magnocellular pathway function contribute to impaired facial affect recognition, however the exact neurobiological background is still unclear.

The ERP components linked to early visual perception and emotion recognition are well studied, however only a few study analyzed the oscillatory correlation of these process^41,42^. Event Related Spectral Perturbation (ERSP) is a measure of spectral power change from baseline, allowing the analysis of the change of EEG signal energy in time in specific frequency bands^43^. In the present study both evoked and induced activity were investigated by calculating the ERSP. This approach gives the possibility to fully understand the electrophysiological activity linked to early visual perception and emotion processing.

Electrophysiological activity within the theta range (4–7 Hz) play an essential role in decoding of facial and emotional information^44,45^. However, in schizophrenia only a few studies analyzed theta activity linked to face and emotion recognition. Decreased theta activity was found in patients with schizophrenia compared to healthy controls over the frontal and central regions in a facial expression recognition study^46^. In our previous investigation we analyzed event-related theta synchronization (ERS, i.e. an increase in theta power) in a facial emotion recognition task, where a significantly decreased theta synchronization in patients with schizophrenia was found^47^. To our knowledge the present investigation was the first to analyze ERS during early-visual perception and ERS during face and emotion recognition tasks and the connection of these processes on the same population of patients with schizophrenia.

Although the magnocellular pathway deficit and impaired emotion recognition have been well documented in schizophrenia, the connection between these domains is still unclear. In this study the connection between the electrophysiological correlates of the perception of magnocellular (LSF, low-spatial frequency)/parvocellular (HSF, high-spatial frequency) biased visual stimuli and facial affect recognition were analyzed.

Due to the deficit of the magnocellular pathway in schizophrenia^48,49^, we hypothesized that event-related theta synchronization to low-spatial frequency (LSF) stimuli would be decreased in schizophrenia patients relative to control subjects, while no similar differences were expected between the two study groups in the high-spatial frequency (HSF) condition, due to the intact parvocellular pathway in schizophrenia.

Based on our previous investigation^47^ we expected decreased theta synchronization in patients compared to healthy controls in face the non-face and in the emotion recognition task.

While no previous investigation examined the relationship of ERS to emotion recognition and ERS to basic visual magnocellular and parvocellular biased stimuli, we did not have one specific hypothesis in this regard. However, we had two competing hypotheses. According to our first hypothesis we expected that ERS in the magnocellular biased condition would be correlated with ERS in the face and facial affect conditions. This hypothesis is in line with the bottom up model of schizophrenia. According to our second hypothesis no such correlation would be found, which may raise the possibility that these two impairments are two distinct endophenotypes of schizophrenia.

## Methods

### Subjects and clinical measures

Thirty-nine subjects with schizophrenia (n = 31) and schizoaffective disorder (n = 8), (24 males, mean age 33.4 ± 10.5 years) and forty healthy control subjects (25 males, mean age 32.8 ± 9.6 years) were enrolled in the study. The two study group were socially matched by gender, age and education (Table 1). All participants were right-handed with the exception of 3 left-handed and 1 mixed handed patient and 3 left-handed and 2 mixed handed healthy controls. All participant had normal or corrected-to-normal vision.

**Table 1:** Demographic information for both study groups and clinical characteristics of the Schizophrenia Group.

	Schizophrenia	Control	statistics	p value
	Group (n = 39) Mean (SD)	Group (n = 40) Mean (SD)		
Gender (male/female)	24/15	25/15	Chi^2^ = 0.008	n.s.
Age	33.4 (10.5)	32.8 (9.6)	t = −0.23	n.s.
Education level*	3/25/11 (8%/64%/28%)	0/28/12 (0%/70%/30%)	Fisher’s exact test	n.s.
Illness duration (years)	8.3 (8.6)	—		
Day Care Unit/In-/outpatient	4/18/17	—		
CPZ equivalent dose (mg)	508.8 (338)	—		
PANSS total score	62.3 (16.4)	—		
PANSS positive subscore	15.2 (4.6)	—		
PANSS negative subscore	16.2 (5.7)	—		
PANSS general subscore	30.9 (8.1)	—		

*Education level: 1 = elementary school/ 2 = high school/ 3 = college/university.

CPZ = chlorpromazine equivalent dose.

PANSS = Positive and Negative Symptoms Scale.

Patient were recruited from Department of Psychiatry and Psychotherapy, Semmelweis University, Budapest, Hungary. All patients met the criteria for schizophrenia based on the Structured Clinical Interview for Diagnostic and Statistical Manual of Mental Disorders, 5th Edition (DSM-V)^50^. Psychiatric symptoms on the PANSS (Positive and Negative Syndrome Scale)^51^ were evaluated by the trained psychiatrist. At the time of the testing all patients took antipsychotic medication, the mean Chlorpromazine equivalent dose^52^ was 508.8 mg/day (SD = 338)^47,53,54^.

The exclusion criteria for patients with schizophrenia were any other DSM-V disorder, any other central nervous system disorder, mental retardation, epileptic seizure, history of head injury with loss of consciousness for more than 10 minutes and alcohol and drug abuse. For healthy controls exclusion criteria were any psychiatric disorder and a global severity index of 114 on the Symptom Checklist-90-R^55^, according to a Hungarian population sample^56^ in order to exclude subjects with high risk for psychiatric disorders^47,53,54^.

Written informed consent was obtained from all the participants/or their legal guardians after a detailed description of the study, which was approved by the Semmelweis University institutional review board. The study was carried out in accordance with the Declaration of Helsinki and all relevant guidelines and regulations.

Demographic information for the study groups and the clinical characteristic of the patients are presented in Table 1.

### Stimuli and procedures

The EEG recording took place in a dimmed, sound-attenuated room. The subjects were instructed to sit in a comfortable chair in front of a table with a computer screen at a distance of about 50 cm. Presentation of all stimulus material and the recording of the given responses was controlled by the Presentation 13.0 software (Neurobehavioral Systems, Inc.; Albany, CA)^47,53,54^.

**Figure 1:**
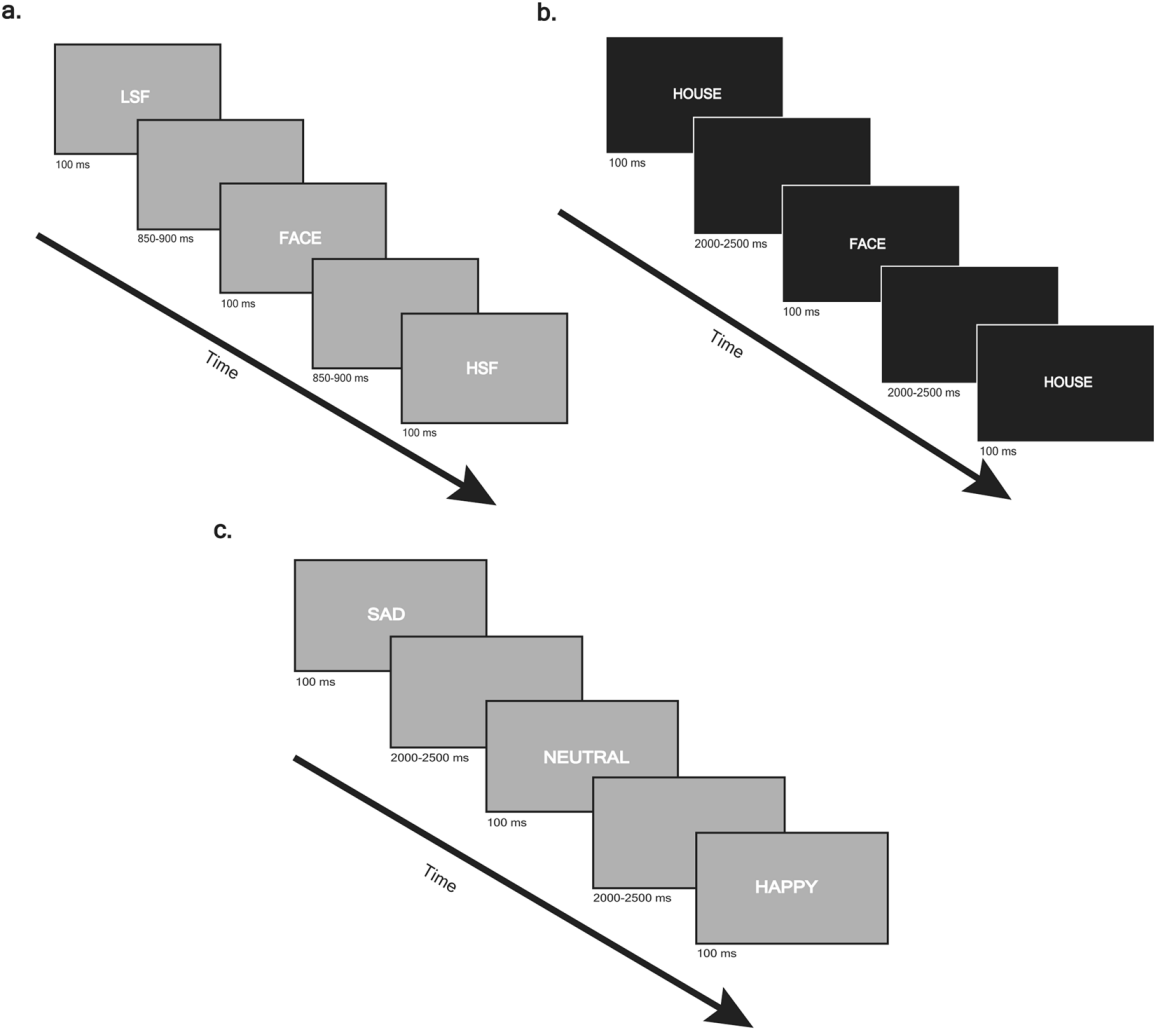
Schematic illustration of the experimental paradigms. (**a**) Visual stimuli paradigm: 224 low-spatial frequency Gabor-patches (p = 0.45), 224 high-spatial frequency Gabor-patches (p = 0.45) and 50 faces (p = 0.1) were presented for 100 ms, the stimulus onset asynchronies were randomized between 850–900 ms. Subjects had to identified faces by button press. (**b**) Face/non-face paradigm: 80 faces (p = 0.5) and 80 houses (p = 0.5) were presented for 100 ms with randomized stimulus onset asynchronies between 2000–2500 ms. The subject had to response by a button press whether they see a face or a house. (**c**) Emotion recognition paradigm: 80 sad (p = 33.3), 80 neutral (p = 33.3) and 80 happy (p = 33.3) faces were presented (from: Karolisnka Directed Emotional Face set: http://www.emotionlab.se/resources/kdef), each for 100 ms, the stimulus onset asynchronies were randomized between 2000–2500 ms. Subjects had to identified emotions by button press.

During EEG recording subjects performed three different paradigms, a visual stimuli paradigm, a face non-face paradigm and an emotion recognition paradigm (Fig. 1).

#### Visual stimuli paradigm

Stimuli were circular gratings sinusoidally modulated with a 2D Gaussian envelope and composed of a fundamental frequency of either 0.8 cycles per degree (Low Spatial Frequency, LSF) or 5 cycles per degree (High Spatial Frequency, HSF)^20^. The stimulus set consisted of 112 low spatial frequency and 112 high spatial frequency Gabor-patches and 25 faces of the Karolinska Directed Emotional Faces^57^. Subjects were asked to identify the faces by button press. Each stimulus was presented for 100 ms with the stimulus onset asynchronies of 850–900 msec. 2 blocks of stimuli were run.

#### Face non-face paradigm

Stimulus displays consisted images of faces^58^ and houses. The stimulus set consisted of 40 different faces and 40 houses. Participants were instructed to press the right button whenever a face and the left button whenever a house was presented. Each stimulus was presented for 100 ms with the stimulus onset asynchronies of 2000–2500 msec. 2 blocks of 80 trial were run.

#### Emotion recognition paradigm

Subject had to identify emotional expressions from photographs of 8 male and 8 female subjects. The pictures were chosen from Karolinska Directed Emotional Face set^57^. Non-facial parts of the faces (e.g. hair, background) were removed from the pictures. There were 3 photographs from each faces (happy, neutral, and sad). Subjects were instructed to press a button indicating whether the face is happy, neutral or sad. Each stimulus was presented for 100 ms with stimulus onset asynchronies of 2000–2500 msec. Altogether 5 blocks of 48 trial were run. Each block contained 16 sad, 16 neutral 16 happy faces.

### EEG recording and processing

EEG was recorded from DC with a low-pass filter at 100 Hz using a high-density 128-channel BioSemi ActiveTwo amplifier^59^. The electrode cap had an equidistant-layout and covered the whole head. EOG electrodes to monitor eye movement were placed above the right and below the left external canthi. Data was digitized with sampling rate of 1024. Built-in and self-developed functions as well as the freeware EEGLAB toolbox^60^ in the Matlab (MathWorks, Natick, MA) development environment were used for subsequent off-line data analyses. EEG was re-referenced to the common average potential and filtered off-line between 0.5 and 45 Hz using zero phase shift forward, and reverse IIR Butterworth filter^53,54,61^.

Epochs from 600 ms pre-stimulus to 600 ms post-stimulus for the visual task and 1400 ms post-stimulus for the face non-face and for the emotion recognition task were extracted from the continuous EEG for further analysis and corrected for the pre-stimulus baseline. The removal of muscle and eyes movement artifacts (detected by EOG) was performed by ADJUST^62^ an ICA (Independent Component Analysis) based automatic artifact detector. Furthermore, epochs with a voltage exceeding ± 100 μV on any EEG or EOG channel were rejected from the analysis. Data from one patient in the face non-face paradigm and two patients in emotion recognition task were excluded due to numerous uncorrectable artifacts^53,61^.

Total (presented) trial number was 224 low-spatial frequency Gabor-patches and 224 high-spatial frequency Gabor-patches in the visual task, 80 faces and 80 houses in the face non-face task and 80 happy, 80 neutral and 80 sad faces in the emotion recognition task. After artifact rejection, the average number of trials in the control group was 212.8 trials (*SD = *19.3) and 199.0 trials (*SD = 16*.*8*) for the LSF and HSF condition; 74.0 (SD = 8.7) and 73.7 (SD = 8.3) for the face and house condition and 71.8 trials (SD = 11.4), 71.8 trial (SD = 12.1), 71.8 trials (SD = 12.0) for sad, neutral and happy condition, respectively. For patients with schizophrenia the mean trial number was 203.9 trials (*SD = 34*.*2*) and 187.7 trials (*SD = 30*.*9*) for LSF and HSF condition; 69.5 trials (SD = 13.9) and 69.2 trials (SD = 15.0) for the face and house condition and 65.7 trials (SD = 15.9), 66.5 trial (SD = 13.9), 67.0 trials (SD = 13.6) for sad, neutral and happy condition, respectively.

**Figure 2:**
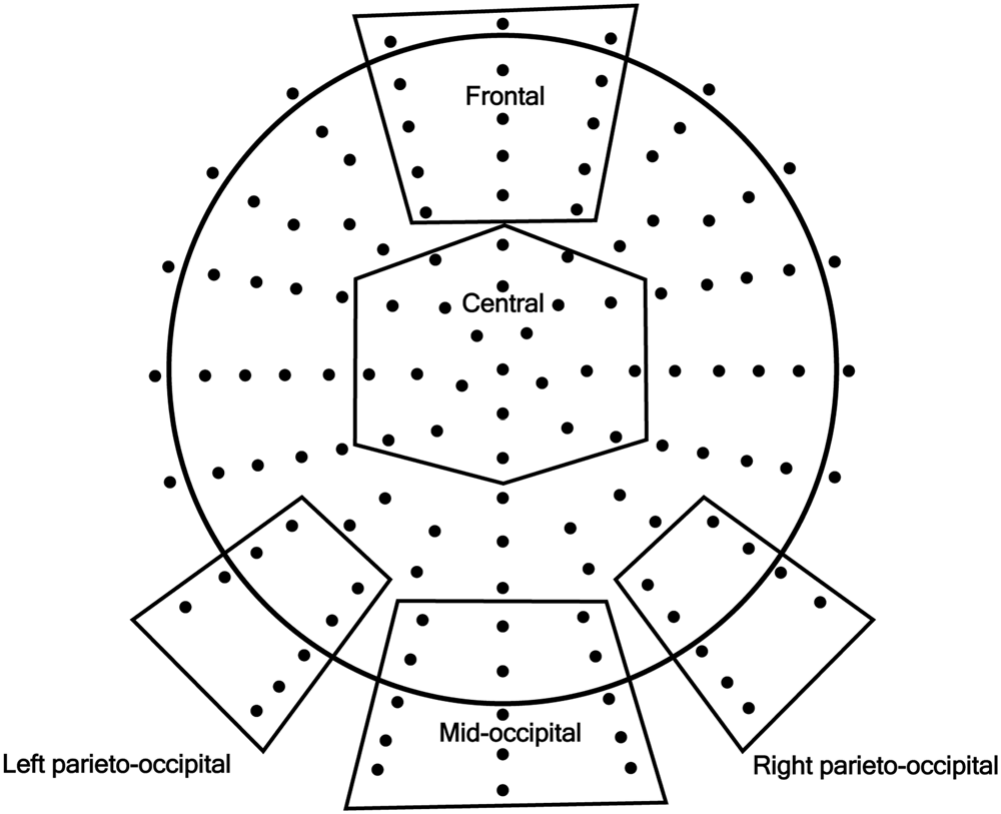
The map of 128 + 2 electrodes and the 5 regions of interest (ROIs): a frontal, a central, a mid-occipital, right parieto-occipital and left parieto-occipital regions. Electrode clusters selected for analyses (Regions of Interests) are marked with black dots in the scalp map.

The 128 channels were divided into 5 regions of interest (ROIs): a frontal, a central, a mid-occipital, and two parieto-occipital regions (Fig. 2). Mean values were calculated by averaging across electrodes within ROIs in order to further attenuate noise.

### Data analysis

Stimulus-related theta (4–7 Hz) activity changes were measured by event-related spectral perturbation (ERSP), that provides a 2-D representation of mean change in spectral power (in dB) from baseline^47,53,61,63^.

To compute the ERSP, baseline spectra are calculated from the EEG immediately preceding each event. The epoch is divided into brief, over-lapping data windows, and a moving average of the amplitude spectra of these is created. Each of these spectral transforms of individual response epochs are then normalized by dividing by their respective mean baseline spectra. Normalized response transforms for many trials are then averaged to produce an average ERSP, plotted as relative spectral log amplitude on a time-by-frequency plane^47,53,61,63^. For further details of the ERSP analysis see the Supplementary Material.

The analysis was performed on epochs extending from 600 ms before to 600 ms after stimulus onset in the visual task and extending from 600 ms before stimulus onset to 1400 ms after stimulus onset in the face/non-face and in the emotion recognition tasks, respectively. The sliding window was 400 ms wide, and it was applied 200 times. No zero padding was applied. The ERSP time-frequency matrices were baseline corrected by the average power calculated from the 600 to 200 ms pre-stimulus period. Dynamical changes in oscillatory activity were studied by computing ERSPs for each trial, then averaging them separately for each condition. Mean ERSP values were calculated by averaging across electrodes within scalp regions to further attenuate noise^47,53,61^.

We selected the time windows for the theta ERS analysis according to the previous ERP and ERSP experiments and the detected peak of the theta synchronization in the three different paradigms.

Based on the detected peak latencies and the previous EEG studies with magnocellular/parvocellular biased stimuli we selected the 140–280 ms time window in LSF condition and the 100–200 ms time windows in the HSF condition for further analysis^18,42^.

Previous electrophysiological experiments indicated a deficit in the structural decoding of faces indexed by a decreased N170 component in patients with schizophrenia in face and facial affect recognition tasks^26,31,64^. A concurrent EEG and fMRI study reported that the fusiform face (FFA) and the sulcus temporalis superior (STS) are associated with the electrophysiological activity of the N170 component^65^ over the right parieto-occipital region, consequently, we analyzed theta ERS over the same region. Theta ERS over the occipito-temporal areas- in the 0–300 ms time window – presumably associates with the facial feature decoding (i.e. N170 component)^66^. Furthermore, in our previous ERSP experiment we found a significant difference in the theta ERS between patients with schizophrenia and healthy controls in the time interval of 140–200 ms^47^. In addition we took into consideration the peak of the theta ERS in the face, non-face task; therefore, we selected the 140–240 ms time window in the face non-face paradigm.

Previous experiments reported synchronized occipital theta oscillations in paradigms with emotional content^67–69^, Balconi & Lucchiari reported theta oscillatory activity associated with emotion recognition in the 150–200 ms time window^44^. Based on their and other emotion recognition studies^47^ and the detected peak of theta ERS the analysis of the emotion recognition task was performed on the 140–200 ms time window.

The different effects on ERSP were tested by three-way analyses of variance (ANOVA) of study group (healthy control (HC) vs. schizophrenia (SZ)) × ROI (a frontal, a central, a mid-occipital, and two parieto-occipital) × stimulus type (HSF vs. LSF or face vs. non-face or sad vs. neutral vs. happy). All the main effects and the 2-way and 3-way interactions are included into the ANOVA model. Since between-group comparisons were evaluated over five regions, Hochberg correction for multiple comparisons was applied to the post-hoc contrasts^47,53,61,70,71^.

The associations of emotion recognition performance with ERSPs were investigated by Spearman correlation, since emotion recognition measures deviated from the normal distribution. For the same reason correlations of CPZ equivalent doses and PANSS scores with ERSPs were also investigated by Spearman correlation^53^.

## Results

### Behavioral results

Behavioral scores deviated from normal distribution, thus non-parametric Mann-Whitney U tests were applied. In the emotion recognition task, the difference between hit rates of controls (mean hit rate = 89.1% SD = 3.7) and patients with schizophrenia (mean hit rate = 80.3%, SD = 10.7) was significant (U = 324, p < 0.001). Reaction time in patients was significantly longer (t = 2.97, p = 0.004). Results of the emotion recognition task are summarized in Table 2.

**Table 2:** Emotion recognition performance (mean (SD)).

	Subjects with schizophrenia (n = 39)	Healthy control participants (n = 40)	Statistics	p value
Total hit rate	80.3% (10.7)	89.1% (3.7)	U = 324	<0.001
Sad hit rate	76.6% (12.1)	85.7% (6.3)	U = 434	<0.001
Neutral hit rate	75.8% (18.6)	88.2% (5.9)	U = 418	<0.001
Happy hit rate	88.8% (10.1)	93.9% (3.1)	U = 554	0.03
Reaction time	783.8 ms (125.9)	714.2 ms (77.3)	t = 2.97	0.004

### The between group comparison of Theta synchronization in visual task

**Figure 3:**
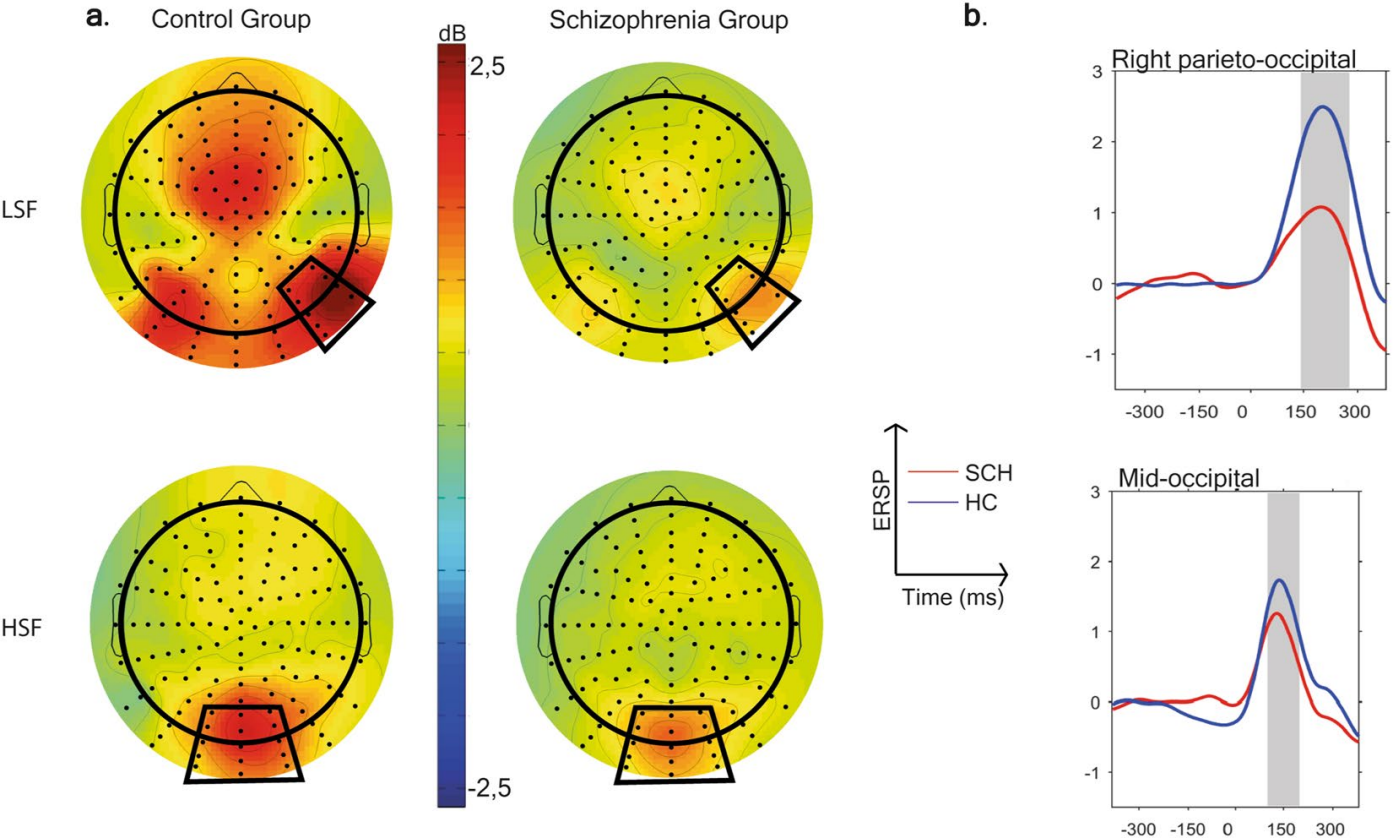
(**a**) Scalp topography of the theta event-related spectral perturbation (ERSP) in the 140–280 ms (LSF) and in the 100–200 ms (HSF) time windows and (**b**) theta ERSP to the low-spatial frequency (LSF) and high-spatial frequency (HSF) conditions in the two study groups (blue = Healthy control participants, red = Subjects with schizophrenia).

In the visual stimuli task an increase in ERSP, in other words a theta synchronization (ERS) was observable to LSF and HSF conditions, in both study groups (Fig. 3).

There was a significant main effect of study group (F(1,77) = 10.87, p = 0.0015) on theta ERS, indicating a decreased theta synchronization in patients relative to controls. Region also had a significant effect on theta ERS (F(4,77) = 20.15, p < 0.0001). A significant main effect of stimulus condition (F(1,77) = 11.45, p < 0.001) was also detected indicating a stronger theta ERS to LSF (t = 3.62, df = 77, p = 0.0005, in right-parieto-occipital region) compared to HSF condition.

**Figure 4:**
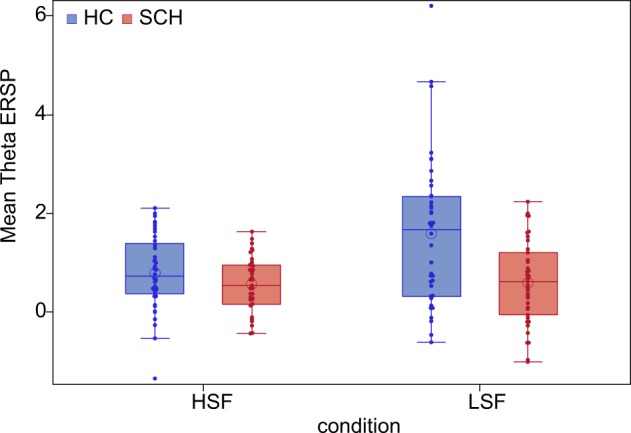
Mean theta ERSP in high-spatial frequency-HSF (time window: 100–200 ms) and low-spatial frequency-LSF (time window: 140–280 ms) conditions in the two study groups. (blue = Heathy control participants, red = Subjects with schizophrenia, box = interquartile range (IQR), dots = measurements on single subjects, error bars = maximum and minimum observation inside the 1.5 IQR, line in the box = median, circle = mean).

The 2-way interaction of study group and stimulus condition was also significant (F(1,77) = 9.65, p = 0.003). This interaction was analyzed further by post hoc t comparisons indicating that theta ERS for LSF stimulus condition was decreased in patients relative to controls (t = 3.59, p = 0.0006), while no similar between group difference was found for the HSF stimulus condition (t = 1.54, p = 0.13) (Fig. 4).

### The between group comparison of Theta synchronization in 140–240 ms time window in face non-face task

**Figure 5:**
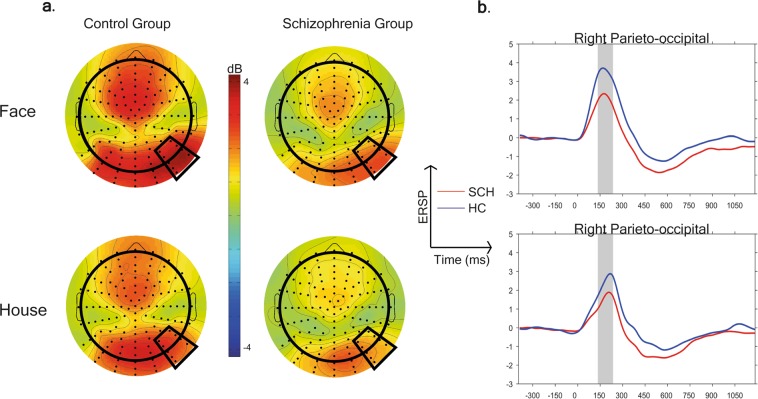
(**a**) Scalp topography of the theta event-related spectral perturbation (ERSP) in the 140–240 ms time windows and (**b**) theta ERSP to face and non-face conditions in the two study groups (blue = Healthy control participants, red = Subjects with schizophrenia).

A theta synchronization (ERS) was also observable to face and house conditions, in both study groups (Fig. 5).

There was a significant main effect of study group (F(1,76) = 6.88, p = 0.011) on theta ERS in the 140–240 ms time window, indicating decreased synchronization in the theta range in patients relative to controls. Region also had a significant effect on theta ERS (F(4,76) = 23.63, p < 0.0001). A significant main effect of stimulus condition (F(1,76) = 20.19, p < 0.0001) was also detected indicating a stronger theta ERS to face compared to house conditions. None of the interactions had a significant effect (p > 0.1).

Effect size in term of cohen’s d (cohen’s d = mean1 - mean2/ ((SD1 + SD2)/2); /1 = face, 2 = non-face/) in the right-parieto-occipital region between conditions (face vs. house) in the control group was 0.41 and in the schizophrenia group 0.29, separately.

After covarying for the LSF - HSF difference in the analysis of the face non-face task the group difference did not remain significant (F(1,75) = 2.71, p = 0.1), while the LSF-HSF difference (F(1,75) = 44.15, p < 0.0001) had a significant effect on theta ERS.

### The between group comparison of Theta synchronization in the 140–200 ms time window in the emotion recognition task

**Figure 6:**
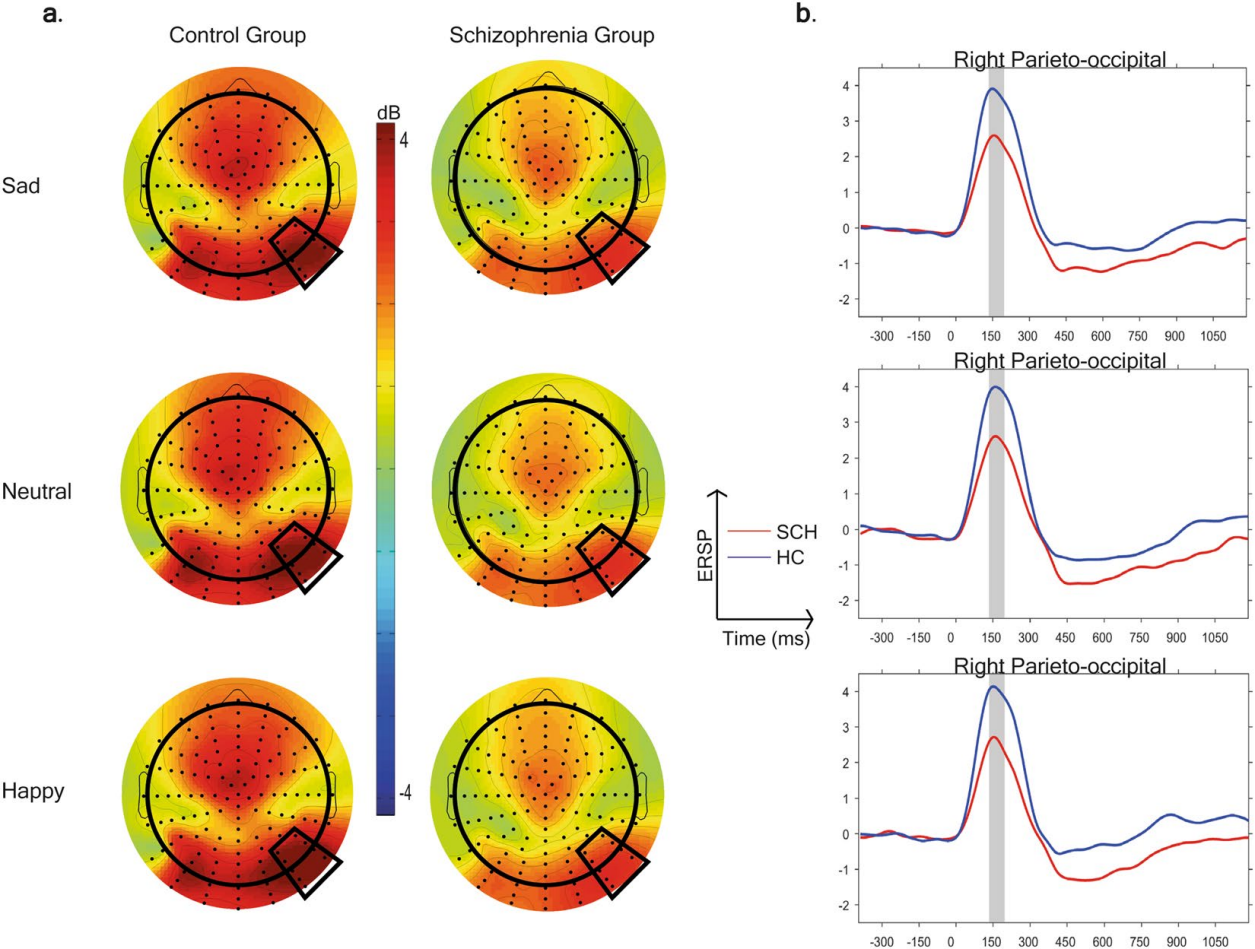
(**a**) Scalp topography of the theta event-related spectral perturbation (ERSP) in the 140–200 ms time window and (**b**) theta ERSP to the three experimental conditions in the two study groups (blue = Healthy control participants, red = Subjects with schizophrenia).

During emotion recognition task theta ERS was observable to all conditions, in both study groups (Fig. 6).

There was a significant main effect of study group (F(1,75) = 8.5, p = 0.0047) on theta ERS in the 140–200 ms time window, indicating stronger synchronization in the theta range in controls relative to patients. Region also had a significant effect on theta ERS (F(4,75) = 29.0, p < 0.0001) with a maximum in the right parieto-occipital region in both study groups.

The main effect of stimulus condition and the 2-way and 3-way interactions were not significant (p ˃ 0.05).

After covarying for the LSF - HSF difference in the analysis of the emotion recognition task the group difference did not remain significant (F(1,74) = 2.99, p = 0.09),while the LSF-HSF difference (F(1,74) = 53.63, p < 0.0001) had a significant effect on theta ERS.

### Correlation between Theta synchronization in visual task and face non-face task

Theta activity in LSF condition (magnocellular biased) correlated significantly with theta activity in face non-face task in control group (face: r = 0.57, p = 0.0001; house: r = 0.59, p < 0.0001) and in schizophrenia group (face: r = 0.42, p = 0.009; house: r = 0.57, p = 0.0002).

In contrast the correlations between theta activity in HSF condition (parvocellular biased) and in face condition were not significant in any study groups (p ˃ 0.05).

### Correlation between Theta synchronization in the visual and in the emotion recognition task

Theta activity in the LSF condition (magnocellular biased stimuli) correlated significantly with theta activity in the emotion recognition task in the control group (sad: r = 0.67, p < 0.0001; neutral: r = 0.61, p < 0.0001; happy: r = 0.67, p < 0.0001) and also in the schizophrenia group (sad: r = 0.62, p < 0.0001; neutral: r = 0.58, p = 0.0002; happy: r = 0.56, p = 0.0003).

In contrast correlations between theta activity in HSF condition (parvocellular biased stimuli) and in emotion recognition were not significant in any of the study groups (p ˃ 0.05).

### Correlation between Theta synchronization in the face non-face and in the emotion recognition task

Theta ERS in the face condition correlated significantly with theta activity in the emotion recognition task in the control group (sad: r = 0.83, p < 0.0001; neutral: r = 0.84, p < 0.0001; happy: r = 0.83, p < 0.0001) and also in the schizophrenia group (sad: r = 0.83, p < 0.0001; neutral: r = 0.75, p < 0.0001; happy: r = 0.83, p < 0.0001).

Theta ERS in the house condition correlated significantly with theta activity in the emotion recognition task in the control group (sad: r = 0.72, p < 0.0001; neutral: r = 0.71, p < 0.0001; happy: r = 0.70, p < 0.0001) and also in the schizophrenia group (sad: r = 0.71, p < 0.0001; neutral: r = 0.63, p < 0.0001; happy: r = 0.65, p < 0.0001).

### Correlation between Theta synchronization and behavioral performance

In the patient group emotion recognition task performance correlated significantly with theta ERS to (magnocellular biased) LSF condition (total hit score: r = 0.35, p = 0.03), but not correlated with ERS to (parvocellular biased) HSF condition (total hit score: r = 0.12, p = 0.45).

Also in the patient group significant correlations were found between emotion recognition task performance and theta ERS in the emotion recognition paradigm in the sad condition (total hit score: r = 0.36, p = 0.03), in the neutral condition (total hit score: r = 0.37, p = 0.02) and also in the happy condition (total hit scores: r = 0.33, p = 0.049). Moreover, theta ERS in face condition (in the face/house task) correlated significantly with the behavioral results (total hit score: r = 0.36, p = 0.03).

In the control group the correlation between emotion recognition task performance and theta ERS in any tasks did not reach the significance (p ˃ 0.05).

### Correlations of Theta synchronization with clinical measures

No correlation was found between theta ERSs and clinical variables such as PANSS scores^51^, antipsychotic doses in term of CPZ equivalents^52^ (p > 0.05).

## Discussion

While an early visual impairment in schizophrenia was also described by Kraepelin in the nineteenth century, the exact neurobiological underpinning of this deficit is still untangled. The present study examined the electrophysiological correlates of early visual perception, face and object (house) perception and emotion recognition and their connection in patients with schizophrenia. Early sensory perceptual processing within the magnocellular/parvocellular pathway was tested with low- (LSF, magnocellular biased) and high-spatial (HSF, parvocellular biased) frequency Gabor-patches, while structural decoding of faces was examined by presentation of faces. Facial emotion recognition was tested with presentation of sad, neutral and happy faces. Behavioral performance in emotion recognition and the electrophysiological correlates of the tasks were compared between patients and socially matched controls.

Impaired facial affect perception, which contributes to poorer social cognition^72^, have been extensively documented in patients with schizophrenia. In this study patients were less accurate and showed a delay in recognizing facial affects across all three emotions compared to healthy controls. These are consistent with previous investigations^26,34^ indicating impaired emotion recognition in schizophrenia. Identification of neutral faces and sad emotions were more difficult for patients than the recognition of happy facial displays, which is also in line with previous results describing a more prominent deficit in negative emotion recognition^22,73^.

Patients in this study showed a decreased theta ERS to magnocellular, but not to parvocellular biased stimuli. This finding is in line with the work of Martinez *et al*.^42^, who also found a reduced theta phase synchrony to stimulus attended and unattended LSF stimuli, but not to HSF stimuli in patients with schizophrenia. Several behavioral^10,11,14^ electrophysiological^16–18^ and fMRI^4^ studies have also demonstrated a magnocellular deficit in patients with schizophrenia.

In the face non-face paradigm face stimuli induced greater theta ERS compared to non-face stimuli in both study groups, which is in line with previous studies showing increased electrophysiological activities to faces compared to non-face objects^74^. Subjects with schizophrenia responded with decreased theta ERS to both facial and non-facial stimuli compared to control subjects. This finding supports our previous results describing decreased event-related theta ERS in patients with schizophrenia relative to healthy controls^47^. Our results, namely that a decreased theta ERS in patients were found not only in the face but also in the non-face condition support the general visual decoding deficit hypothesis in schizophrenia^33,34^. An event related potential (ERP) analysis was also performed, but no face specific between group difference was found in the N170 component (Supplementary Material). However several previous studies showed face and facial affect specific impairments in patients with schizophrenia^64,75,76^. These results taken together with our findings support the notion that there is a general visual deficit in schizophrenia which may contribute to the specific impairment seen in facial expression and emotion recognition^77^.

In the emotion recognition task a decreased theta ERS was found to all emotion conditions in the patient group relative to controls. This finding is consistent with our previous results, where decreased event-related theta synchronization was detected in patients in the same time window (140–200 ms) and with a similar scalp distribution^47^. Nevertheless, no difference in theta ERS between emotion conditions was detected in this paradigm. Also the ERP analysis of the N170 and N250 components did reveal any emotion specific differences between study groups (Supplementary Material). The possible explanation might be, that only details of the faces are being processed in this early time window and the exact emotion processing appears later^53^, so the deficit in the visual perception occurs before the emotion processing. This notion is supported by the work of Knyazev and colleagues, who found that implicit emotion processing of faces were associated with early (before 250 ms) theta ERS, while explicit emotional content processing associated with late (after 250 ms) theta synchronization in healthy subjects^45^.

The significant group differences in theta ERS disappeared after covarying for the LSF - HSF difference both in the face non-face task and in the emotion recognition task. Based on this result it seems that the magnocellular deficit drives the differences in higher level functions – such as face-, non-face- and emotion recognition. Thus, the deficit in the early stage visual perception lead to higher level process impairments.

In the ERP analyses no significant between-group differences were detected in the N170 and N250 components while significant group differences were found in theta ERS in the same time periods. This discrepancy could be explained by the difference between the two techniques: the conventional ERP technique could give only a partial insight into the electrophysiological process, because it reflects only phased-locked evoked activity and the induced activity is not phase-locked to the stimuli, therefore these potentially important induced activity will be averaged out. In contrast, ERSP technique captures total power including both evoked and induced activity^78^.

Furthermore, theta ERS in the magnocellular biased (LSF) condition but not in the parvocellular biased (HSF) condition showed significant correlation with theta ERS to face, to non-face (house), and also to emotional face stimuli in both study groups. Furthermore, theta ERS in the face and in the non-face conditions showed correlations with theta ERS in the emotion recognition task in both study groups. The processes behind the recognition of objects can be explained by the “frame and fill” model; the information delivered rapidly by the magnocellular pathway – via the dorsal stream - creates a low-resolution templates of the object in the frontal brain areas, than it gives a feedback to the ventral temporal cortex, which then filled in with detailed information by the much slower parvocellular pathway^79,80^. The two main visual pathway provide different information about the details of the face; the magnocellular pathway provides information about the global configuration, the shape of the face, and emotional cues, while the parvocellular pathway gives the information about the fine details of the faces^32,81^. Based on our results it seems that global information is also vital for correct object, face and emotion recognition. This notion is also supported by the work of Calderone^82^, who studied the contributions of magnocellular (LSF) and parvocellular (HSF) information processing to the impaired object recognition in schizophrenia by fMRI. In line with our results, they found a LSF biased stimuli processing (magnocellular) impairment in schizophrenia. Furthermore, they found a decreased activation in the primary visual cortex in the dorsal stream, and in the frontal and ventral temporal cortex to magnocellular biased (LSF) objects in the patients group. In sum our findings lend support to the notion by recent reviews, that altered object recognition and impaired face/facial affect recognition in patients with schizophrenia are both caused by early sensory deficits in the magnocellular pathway^77,83^.

In the patient group a worse performance in the emotion recognition task was also associated with decreased theta ERS in the magnocellular (LSF) condition, while no similar association was found between emotion recognition and theta ERS to parvocellular (HSF) condition. Also a decreased theta ERS to face, non-face and emotional stimuli were associated with a decreased emotion recognition performance in patients but not in controls. These findings also support the notion that a magnocellular deficit contributes to higher level functioning impairments. The lack of correlation between ERS and emotion recognition performance in the control group might be explained by a smaller variance in magnocellular functioning and emotion recognition performance in healthy subjects.

In sum our findings suggest that in the patients with schizophrenia the early visual perception dysfunction may play a critical role not only in the general perception of objects but also in the emotion recognition deficit. Thus, our results further support the growing evidence to the bottom-up model of disrupted cognition in schizophrenia, which indicate that early sensory deficit contribute to the impaired higher level dysfunction^14,15,33,34,38,40,82,84^.

There are some possible limitations of this study. First, we applied different photos of faces in the face non-face paradigm and in the emotion recognition task. Second, all patients were on medication during testing. However, no significant correlations were found between chlorpromazine equivalent doses and theta synchronization in any paradigm. The EEG experiment was long and complex, hence we included patients with good compliance and thus symptom severity scores were in the low/medium range (mean PANSS total score was 62.3). It might have caused a lack of correlation between theta ERS and symptoms scores.

## Conclusions

In this study we found that patients with schizophrenia show decreased magnocellular function relative to healthy controls and this deficit correlated with impaired affect recognition performance, and also correlated with the electrophysiological correlates of face and emotion recognition. Overall, our findings suggest that the deficit in magnocellular pathway contributes to impaired face and facial affect recognition in patient with schizophrenia, which finding gives further support to the bottom-up model of disrupted face perception and emotion recognition in schizophrenia.

## Supplementary information

Supplementary information, From basic perception deficits to facial affect recognition impairments in schizophrenia

## Data Availability

The datasets that are used and/or analyzed during the current study are available from the corresponding author on reasonable request.
