# Supplementary material for: From basic perception deficits to facial affect recognition impairments in schizophrenia
Source: Sci Rep. 2019 Jun 20;9:8958. doi: 10.1038/s41598-019-45231-x (PMC6586813; doi:10.1038/s41598-019-45231-x)
Supplement: Supplementary file 1 — Supplementary information, From basic perception deficits to facial affect recognition impairments in schizophrenia [file 41598_2019_45231_MOESM1_ESM.pdf]

Supplementary information

From basic perception deficits to facial affect recognition impairments in schizophrenia

Csilla Marosi, MD[a], Zsuzsanna Fodor, MD[a], Gábor Csukly, MD, PhD[a,*]

[a] Department of Psychiatry and Psychotherapy, Semmelweis University, Budapest, Hungary

[*]Corresponding Author (address: 1083, Balassa 6, Budapest, Hungary)

Email: csukly.gabor@semmelweis-univ.hu, csugab@yahoo.com;

Tel.: +36-20-825-0174; Fax.: +36-1-210-0336

Details of time-frequency analysis

The method described here generalizes the narrow-band measures of event-related synchronization and desynchronization introduced by Pfurtscheller and Aranibar [1] and includes both phase-locked and non-phase-locked contributions.

The principle of calculating the ERSP is to compute the power spectrum of the EEG signal from a sliding time window. For n trials, if $F_k(f,t)$ is the power of trial k at frequency f and time t, the ERSP value is calculated as

$$ERSP(f,t) = \frac{1}{n}\sum_{k=1}^{n} | F_k(f,t) |^2$$

In order to obtain the $F_k(f,t)$ function (the signal power at a given frequency and time point), the EEG signal was convolved with Hanning-windowed sinusoidal wavelets. The number of wavelet cycles increased evenly with frequency (starting at 0.2 cycles at 0.3 Hz) for optimal time-frequency resolution.

Deatails of Event-related potential (P100, N170, N250 component) analysis

Methodes

We selected the 100-140 ms time window in the visual task and in the face non-face paradigm and 90-110 ms time windows in the emotion recognition task to analyze P100 reflecting the early visual perception. The analyzed second component was N170, which linked to structural decoding of the face and we analyzed it in the 160-180 ms time window in the face non-face paradigm and 145-170 ms time window in the emotion recognition task. In the emotion recognition task we further analyzed the early affect modulation by N250 component in the 210-230 ms time window.

As in the ERSP analysis, the 128 channels were divided into 5 regions of interest (ROIs): a frontal, a central, a mid-occipital, and two parieto-occipital regions (see Supplementary Figure S1). Mean values were calculated by averaging across electrodes within ROIs in order to further attenuate noise.

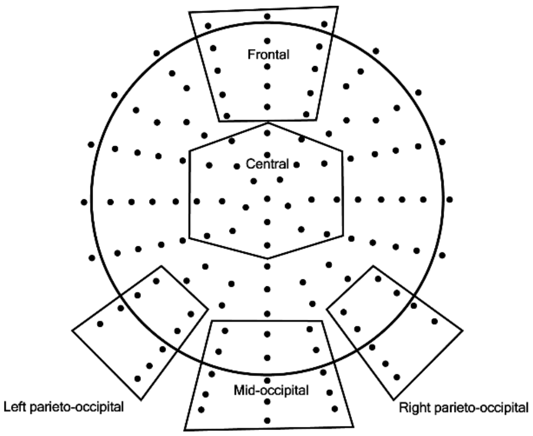

The different effects on Event-related potentials (ERPs) were tested by three-way analyses of variance of study group (healthy control (HC) vs. schizophrenia (SZ)) × ROI (a frontal, a central, a mid-occipital, and two parieto-occipital) × stimulus type (HSF vs. LSF or face vs. non-face or sad vs. neutral vs. happy). All the main effects and the 2-way and 3-way interactions are included into the ANCOVA model.

Post-hoc pairwise contrasts were conducted to investigate the interactions. Since between group comparisons were evaluated over 5 regions, Hochberg correction for multiple comparisons was applied to post-hoc contrasts [2,3].

Results

The between group comparison of P100 in visual task

The P100 component did not differ significantly between study groups ($F(1,77) = 2.13$, $p = 0.15$). A significant main effect of stimulus condition ($F(1,77) = 122.56$, $p < 0.0001$) was detected (LSF: 0.59 ± 1.9 μV, HSF: 0.08 ± 1.5 μV). Region had a significant effect on P100 ($F(4,77) = 28.32$, $p < 0.0001$), with maximum P100 amplitude the LSF condition in the mid-occipital region in both study groups. The two-way interaction of study group and region ($F(4,77) = 3.02$, $p = 0.023$), and region and stimulus condition ($F(4,77) = 52.45$, $p < 0.0001$) were significant.

The two-way interaction of study group and stimulus condition was not significant ($F(1,77) = 0.58$, $p = 0.45$), nor the three-way interaction of study group, region and stimulus condition ($F(4,77) = 0.2$, $p = 0.94$).

The between group comparison of ERPs in face no-face task

P100

The P100 component did not differ significantly between study groups ($F(1,76) = 0.65$, $p = 0.42$). A significant main effect of stimulus condition ($F(1,76) = 26.63$, $p < 0.0001$) was detected (face: 0.89 ± 3.4 μV, house: 1.08 ± 3.7 μV). Region had a significant effect on P100 ($F(4,76) = 36.36$, $p < 0.0001$), with maximum P100 amplitude in the right parieto-occipital region in both study groups. The two-way interaction of region and stimulus condition ($F(4,76) = 7.45$, $p < 0.0001$) was significant.

The two-way interaction of study group and stimulus condition had no effect on the P100 component ($F(1,76) = 0.64$, $p = 0.43$), nor was the study group and region two-way interaction ($F(4,76) = 0.39$, $p = 0.82$) and the three-way interaction of study group, region and stimulus condition ($F(4,76) = 0.53$, $p = 0.71$).

N170

The N170 component did not differ significantly between study groups ($F(1,76) = 0.56$, $p = 0.46$). A significant main effect of stimulus condition ($F(1,76) = 378.25$, $p < 0.0001$) was detected (face: -0.82 ± 4.2 μV, house: 0.43 ± 3.6) . Region had a significant effect ($F(4,76) = 9.43$, $p < 0.0001$), with maximum N170 amplitude in the face condition in the right parieto-occipital region in both study groups. The two-way interaction of region and stimulus condition ($F(4,76) = 115.24$, $p < 0.0001$) was also significant.

The two-way interaction of study group and stimulus condition was not significant ($F(1,76) = 3.51$, $p = 0.06$), nor was the study group and region two-way interaction ($F(4,76) = 1.28$, $p = 0.28$) and the three-way interaction of study group, region and stimulus condition ($F(4,76) = 1.88$, $p = 0.12$).

The between group comparison of ERPs in emotion recognition task

P100

The P100 component did not differ significantly between study groups ($F(1,75) = 0.43$, $p = 0.51$). Region had a significant effect ($F(4,75) = 9.43$, $p < 0.0001$), with maximum P100 amplitude in the right parieto-occipital region in both study groups. While the main effect of stimulus condition and the interactions were not significant ($p > 0.05$).

N170

The N170 component did not differ significantly between study groups ($F(1,75) = 2.49$, $p = 0.12$). A significant main effect of stimulus condition ($F(2,75) = 6.48$, $p = 0.0025$) was detected (neutral vs. happy: $t = 3.6$ $df = 75$, $p = 0.0006$; neutral: -1.27 ± 4.5 μV and happy: -1.38 ± 4.7 μV) . Region had a significant effect on N170 ($F(4,77) = 34.64$, $p < 0.0001$), with maximum N170 amplitude in the right parieto-occipital region in both study groups. The two-way interaction of condition and stimulus region ($F(8,75) = 4.12$, $p = 0.0004$) was significant.

The two-way interaction of study group and stimulus condition had no effect on the N170 component ($F(2,75) = 0.38$, $p = 0.69$), nor was the study group and region

two-way interaction ($F(4,75) = 0.47$, $p = 0.75$) and the three-way interaction of study group, region and stimulus condition ($F(8,75) = 1.21$, $p = 0.31$).

N250

The N250 component did not differ significantly between study groups ($F(1,75) = 0$, $p = 0.96$). A significant main effect of stimulus condition ($F(2,75) = 14.44$, $p < 0.0001$) was detected (sad vs neutral: $t = -4.9$ $df = 75$, $p < 0.001$ and neutral vs happy: $t = 4.5$ $df = 75$, $p < 0.001$; sad: 0.99 ± 4.1 µV and neutral: 1.15 ± 4.3 µV and happy: 1.02 ± 4.2 µV). Region had a significant effect on N250 ($F(4,75) = 29.76$, $p < 0.0001$)), with maximum N250 amplitude in the frontal region in both study groups. The two-way interaction of region and stimulus condition ($F8,75) = 6.8$, $p < 0.0001$) was significant.

The two-way interaction of study group and stimulus condition had no effect on the N250 component ($F(2,75) = 0.11$, $p = 0.89$), nor was the study group and region two-way interaction ($F(4,75) = 0.6$, $p = 0.67$) and nor was the three-way interaction of study group, region and stimulus condition ($F(8,75) = 0.63$, $p = 0.75$).

References

1 Pfurtscheller, G. & Aranibar, A. Event-related cortical desynchronization deftected by power measurements of scalp EEG. *Electroencephalography and Clinical Neurophysiology* **42**, 817-826, doi:10.1016/0013-4694(77)90235-8 (1977).

2 Hochberg, Y. A sharper Bonferroni procedure for multiple tests of significance. *Biometrika* **75**, 800-802, doi:10.1093/biomet/75.4.800 (1988).

3 Hochberg, Y. & Benjamini, Y. More powerful procedures for multiple significance testing. *Stat Med* **9**, 811-818 (1990).

Figure Legends

The map of 128 + 2 electrodes and the 5 regions of interest (ROIs): a frontal, a central, a mid-occipital, right parieto-occipital and left parieto-occipital regions. Electrode clusters selected for analyses (Regions of Interests) are marked with black dots in the scalp map
